# False Impressions

**Published:** 1889-03-09

**Authors:** Caroline Fothergill

**Affiliations:** Author of "An Enthusiast," "A Voice in the Wilderness," etc.


### FALSE IMPRESSIONS.

By CAROLINE FOTHERGILL, Author of "An Enthusiast," "A Voice in the Wilderness," etc.

*(Continued from p. 352.)*

### CHAPTER XXII.—HOLIDAY TIME.

MRS. LEDWARD was very unhappy. We know she did not sympathise with Beatrice's philanthropic, or, as she herself called them, social schemes. She thought that, on the whole, things might very well be left as they were, or if any changes became desirable and necessary, they might be effected without her having to take any share in them. Although she never dared to say so to Beatrice, she had a natural dislike to people lower in the social scale than herself. She was unable to get on the same ground with them; she did not know what to talk to them about; her usually fluent tongue was tied in their presence. Their unfettered manners and customs, and rough, frank speech, were horrible to her, and she had a difficulty in understanding any speech not uttered in "high English." This being so, the two months of August and September had been a long-drawn misery to her. She had first desperately tried to do her duty and stick to her post, and after she had failed ignominiously, and retired from the field, she had been tortured by the pangs of a guilty conscience, which made her feel that she had shown herself a thorough moral coward. This is what had happened.

The very day after they had dined at George Murray's, Beatrice had announced her intention of going from home again, and had furthermore revealed the subject of her meditations when they had been away earlier in the summer. She said that while she had been enjoying the exquisite freshness and beauty of the country, her thoughts had been very much drawn towards her working friends in Mayfield, who were shut out from all but the scantiest participation in such delight, and she had been revolving a scheme for getting them a breath of country air.

"Are you going to give them a treat?" asked Laura. "Shall you organise a cheap trip, or a tour? I hope you are not thinking of personally conducting it. Who will negotiate with the railway authorities? Shall you ask Mr. Murray to help you? If you do it yourself, you are sure to be cheated."

"My scheme scarcely concerns the railway people at all," answered Beatrice. "It took me a long time to think it into shape, but it is all ready now, and I can carry it out whenever I like."

"Let me hear it," said Laura; "I am curious about it."

"You remember my telling you of that large house that was to let at Limehill, and we went over afterwards and saw it?"

"I remember you said it would exactly suit your purpose, but as you did not reveal what that purpose was, I was no wiser than before."

"I took it for two months," went on Beatrice, "and I am going there the day after to-morrow. During those two months we shall have nine Saturdays and Sundays—'week-ends' they call them here. On every one of those Saturdays and Sundays, I intend to have as many of the working people whom I know here to stay with me as the house will hold."

"Beatrice!" ejaculated Laura, too thunderstruck to say any more.

"I know you won't like it, and I am sorry, but I intend to have them all the same. I have thought it well over, and have drawn up a list of the people I am going to invite. I am going to write to-night and invite the first party for Saturday."

"How many will there be?"

"Six. If some can't come, others will."

"And how are you going to do it? Shall you pay their fares?"

"Of course not; that would not be at all the kind of thing I mean. The fare there and back, third class, is half-a-crown. All the people I know can afford that, but it might not be convenient to them to stop all night at an inn, and so they hurry back the same day, or do not come out at all. They will come out to me by a certain train on Saturday afternoon and go back on Sunday evening. The railway people have very kindly consented to stop their Sunday evening express at Limehill during the next two months to pick up my visitors."

"Alas, that wealth can do so much!" groaned Laura. "I never wished that you were poor before. Why do you limit yourself to six? Why not pack the whole lot in at once and have hem all done with at one time?"

"Because I want to give them each a room, and entertain them in decency, as I do my other friends. And I don't want to get rid of them all at once. I want them to enjoy themselves, and also to enjoy myself."

"You won't enjoy yourself," said Laura, with quick scorn. "I don't see how you can. What will you do with them?"

"If it is fine, we shall take walks and drives, and explore the country. If it is too wet to go out, we will arrange amusements indoors."

"Arrange!" scoffed Laura. "Yes, I know that kind of arranging. It will be a perfect nightmare as long as it lasts."

"I shall not trouble you with it," said Beatrice. "Indeed, if you so much dislike the idea, I hope you will not come with me. Stay here, or go and amuse yourself elsewhere; it would considerably take from my pleasure to have you endure a martyrdom for my sake."

"Oh, no," said Laura, with an expression of heroic constancy, which seemed perfectly ludicrous to Beatrice under the circumstances. "I will stand by you and see you safely through it all. But, all the same, I wish you would not take up such crazes; they are very embarrassing to sane and ordinary people. What put such a notion into your head?"

"I thought of it," said Beatrice, quietly. "I have a great deal of money—far more than I should ever feel inclined to spend on myself, and you know that just giving away things does not satisfy me. I always think it is shirking one's responsibility one ought to do as well. I can make friends with people who don't live just as I do, and I have made a great many such friends. I care for them a great deal more than for many of the rich people I know. I can't go away in this beautiful summer weather and enjoy myself, and leave the people I call my friends to weary in this great, hot, dusty town. They can't get away for long at a time, or to a great distance, so I am going as near as I can to be in the pure country, and I shall invite them to come out and stay with me. It will be very easy and simple. I wish more people would do it."

"Well," said Laura, crushed, but not convinced, "I think you are very queer. If you invited the very poor, and gave them a treat—just a day in the country—I could understand it; but you always take up the people who seem quite well able to take care of themselves."

"I dare say there is something wrong about me," said Beatrice, "but, to be quite honest, I can't take an interest in those very poor, low people. I can t understand them. The position of Lady Bountiful is one I cannot occupy. It is offensive to me to be always supplying elementary wants, and getting fawned on in return. When it is a matter of doling out to the destitute, and having one's hand kissed with tears of gratitude, I must retire into the background. I can't bear to see human beings any way except upright before one another. There are numbers of people who enjoy doing those things, and I am willing to help them with money as much as I can, but my own personal work must be among people with whom I can get on the same ground. There are numbers of them, intelligent working men and women, to whom a couple of days in the country will be a far greater treat than it could be to those people you were suggesting. I may be mistaken, but that is how I see it, and I must go to work in my own way."

"In that last sentiment I most emphatically agree with you," said Laura, with a little edge in her voice. "I know it would not be of the slightest use arguing with you, and I shan't attempt it; but my opinion is not in the least changed."

Beatrice carried out her scheme, and found it thoroughly successful. There was friendly and congenial intercourse between hostess and guests, and it would be difficult to say which got the most enjoyment out of these summer "week ends." Beatrice was at her very best; the society of these working folk always seemed to bring out the very best that was in her. The "grand manner," which rebuffed many of her friends, and simply petrified people like Edith Lester; the imperious temper, which caused even Mrs. Ledward to sometimes find life with her very trying, vanished utterly, and she was only a charming and happy woman, with just as much dignity as befitted her position.

The weather was perfect, and those nine gatherings were spent almost entirely out-of-doors. The six guests and Miss Stanford rambled all over the country, and as far as botany, geology, and natural history were concerned, she probably got as much information as she herself imparted on other subjects.

Little incidents occurred which filled her with unspoken affection for her friends. In the course of conversation, things came out; admissions, traits of character—quaint enough many of them—instances of self-sacrifice, taken as a matter of course, all tending to confirm her belief that, in choosing these people for her friends and companions, she had, spiritually, done a very good thing for herself.

But Laura could not bear it. She never joined the party except at meal-times, and then she felt thoroughly out of place. One day, too, one of the men, in talking to her, paid her a compliment, quiet and in perfect good taste, but with a point of humour in it which made all the others, including Beatrice, laugh. Laura, in spite of her love of amusement, was very sensitive where she herself was concerned. She could not bear the laughter, and after drawing herself up with dignity, and making a sharp retort, kept silence for the rest of the meal, thereby throwing a chill over the party which not all Beatrice's efforts could dispel. After bearing with her friend's fad for three weeks, she told Beatrice with some regret that she could endure it no longer, and she would be glad if she might be spared every week end. She said she would go home and keep an eye on the house until the return of its mistress.

The first week or two she was dismally dull. Everyone was away, and the town was in the possession of Philistines from the country. Although she intrenched herself behind the high garden walls, she felt very unhappy—for this is the point we have reached in our story. She had deserted Beatrice, and her conscience would not let her rest. Besides this, she was alone, and being by nature gregariously inclined, this was a great trial to her; although she told herself sternly it was no more than she deserved for having treated Beatrice as she had done.

One Saturday, two weeks before Miss Stanford's return, as she was driving home from the station, she saw Edith Lester walking along the street. She stopped the carriage, and sent the proud footman, to his great disgust, careering after the girl, with orders to bring her to the carriage at once. When she came, she promptly ordered her to get in beside her, and drive home and spend the day with her. Edith more honoured than delighted by the invitation, demurred on the ground that she had not her aunt's permission so to dispose of her time. Mrs. Ledward made very short work of this objection. Seeing they were not far from Queen Street, she insisted upon Edith joining her, and then drove round to get Mrs Lester's permission for Edith to come with her. She gained her end, as usual; permission was granted, though not very readily, and Edith was whirled away feeling a little alarmed at the prospect before her.

In spite of her apprehensions, she enjoyed herself. Mrs. Ledward knew by this time how to manage her, and was very kind. She encouraged Edith to talk about herself and George, and the girl desired nothing better. In a very short time Mrs. Ledward was in full possession of all the details of the journey to Switzerland. Edith inadvertently betrayed a good deal more than she had any idea of, both of her aunt's ideas and wishes and her own, and Mrs. Ledward was not long in unravelling something very near the truth from it all. She did not blind herself for a moment to the facts of the case. She saw the depth of Edith's feeling for George, but she hardened her heart, looked at things in the light of reason and common sense, which with her meant always that the weakest must go to the wall, and the more uncomplainingly they accepted their lot, the better; and with her natural celerity she made up her mind what she would do.

*(To be continued.)*

